# The Wear Responses of the Welded Joints of ASTM A335 Gr. P11 Steels Affected by Accelerated Flow Corrosion

**DOI:** 10.3390/ma12213630

**Published:** 2019-11-04

**Authors:** Javier Montero, Almudena Filgueira, Ana García-Diez, José Luís Mier, Carolina Camba

**Affiliations:** 1 Department of Naval and Industrial Engineering, Escuela Politécnica Superior, University of A Coruña, 15403 Ferrol, Spain; javier.lmontero@udc.es (J.M.); jose.mier@udc.es (J.L.M.); carolina.camba@udc.es (C.C.); 2 Chemistry Department, Escuela Politécnica Superior, University of A Coruña, 15403 Ferrol, Spain; almudena.filgueira.vizoso@udc.es

**Keywords:** wear, welding, hardness, accelerated flow corrosion

## Abstract

This study shows the effects of wear on welded joints of ASTM A355 Gr. P11 “Seamless Ferritic Alloy-Steel Pipe for High Temperature Service” steels subjected to the welding procedures established by codes B31.1 and ASME III. The standard welding procedure establishes the following steps: a preheating process, welding and post-weld heat treatment. This generates a wear behavior that depends on the thermal cycles to which the different areas of the joint are subjected. The objective of this article was the study of the behavior against the flow-accelerated corrosion of the welded joints of a low alloy steel. There is the possibility of establishing welding procedures other than those established, while maintaining the safety ranges, depending on the field of application for the steel.

## 1. Introduction

In power plants, steam pipes are subject to conditions of use that imply the occurrence of the flow accelerated corrosion (FAC) phenomenon. One of the most important aspects is to analyze the wear that occurs in them.

Generally, carbon and low alloy steels are used in the manufacture of these pipes, especially in reactor cooling systems in pressurized water reactors’ (PWRs’) auxiliary systems, such as the one used for the removal of residual heat, and steam pipes. The reasons that support the use of this type of alloy are the combination of low cost, good mechanical resistance, an acceptable corrosion resistance in many environments, and service response [1].

Within steam pipes, welded joints are the weakest points of the system and the zones where failures happen most frequently. Different zones can be distinguished due to the influence of the chemical compositions of the steel and the filler metal, and the temperatures reached during the welding process [2,3,4,5]. These zones are the base metal (BM), heat affected zone (HAZ), and fusion zone (FZ). Because of this, differences in the hardness values in the different zones are obtained [6,7]. This hardness variation results in different wear rates caused by the steam flow, which can accelerate the corrosion inside the pipes [8,9,10]. The FAC is clearly influenced by the content of Cr, and when it exceeds 0.1%, the steel resistance to this phenomenon increases [11,12].

This work has focused on the study of A 335 Gr. P11 Steel, a low carbon steel alloyed with Cr and Mo, widely used in the pipes of power generation plants. The wear resistance of these three zones in a welded joint with the standard procedure was evaluated, since variations in the microstructure of the material alter its tribological properties [13]. In addition, joints made with two alternative welding procedures were studied. For that, pin on disk tests were carried out to determine the wear resistance according to ASTM G-99 “Standard test method for wear testing with a pin on disk apparatus” [14]. The study was completed with the determination of hardness and the metallographic analyses of all samples. Finally, an alternative to the standard welding procedure that improves the response to the FAC was proposed.

## 2. Materials and Methods

### 2.1. Material Tested

The steel under study, ASTM A335 Gr. P11 “Seamless Ferritic Alloy-Steel Pipe for High Temperature Service,” is used in the steam circuits of power plants. In this case, it has a thickness of 9.2 mm, and a composition determined by the steel supplier, which is presented in Table 1.

According to the expression from the International Institute of Welding, the carbon equivalent of the steel would be:

(1) $$\begin{array}{c} C.E.=\%C+\frac{\%Mn}{6}+\frac{\%Cr+\%Mo+\%V}{5}+\frac{\%Ni+\%Cu}{15}=0.14+\frac{0.30}{6}+\frac{1.2+0.5}{5}\\ \;=0.5 \end{array}$$

**Figure 1:**
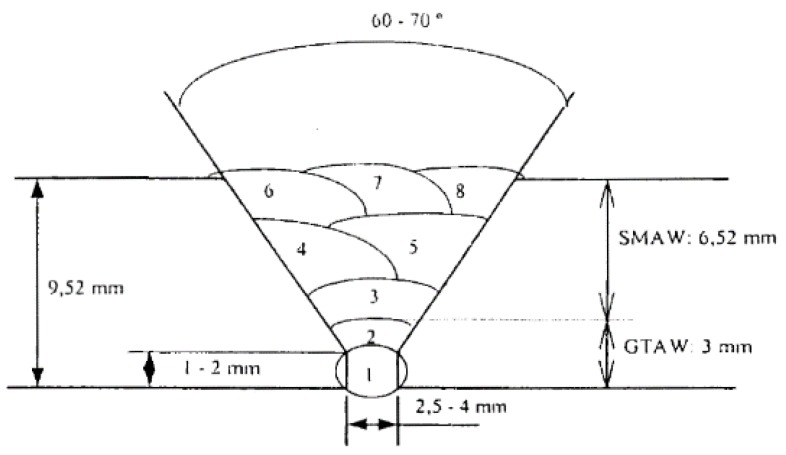
Welding configuration.

Figure 1 presents a diagram of the welding configuration.

The welding procedures that establish the codes and that are used for these types of steels [15,16,17,18,19,20] are the following (code B31.1, and code ASME section III):

- Preheating with a temperature around 100 °C.
- Welding with tungsten inert gas (TIG) in the root with a BOHLER AWS A5.28-96:ER80S-G and using an electric arc welding rod and manual weld filling. (Table 2).
- Post-weld heat treatment (PWHT) with a temperature around 700 °C.

The three subsequent samples show the adjustments (to the welding procedure determined by the standards) that we tested:

- Sample A. Preheating process and post-welding heat treatment, as indicated in the regulations.
- Sample B. Preheating process and elimination of the PWHT.
- Sample C. Elimination of preheating and the PWHT.

Samples were subjected to hardness and wear tests. The data were obtained for the different welding schemes in the three differentiated zones: BM, HAZ, and FZ.

### 2.2. Experimental Work

The study of wear was carried out by a pin in disk test without lubrication, according to the ASTM G99 ‘Standard test method for tests of wear with a pin-in-disk apparatus’ on a Microtest tribometer. Samples with a profile below 0.1 µm were tested to avoid a negative influence of the surface roughness, [14,21]. The test parameters were:

- Applied load of 10 N;
- Rotation speed of 200 rpm with a radius of 3 m;
- Temperature of 200 °C;
- Duration: 1 h;
- Pin: chrome steel ball with a hardness of 775 HV and a diameter of 4 mm.

After the end of the wear test, the mass loss of the sample was determined, subtracting the weights before and after the pin on disk test. Subsequently, Vickers microhardness tests were performed for each of the welding zones. The test parameters were:

- Test load: 200 g;
- Application time: 20 s.

Finally, the metallographic analysis of the areas to be studied in each of the samples (untreated steel, thermally affected area and welding) was performed. The samples were attacked with 3% Nital. The study was completed with energy-dispersive X-ray spectroscopy (EDS) to identify the different phases present in the microstructure.

## 3. Results and Discussion

The hardness tests performed for each procedure (samples A, B, and C) and zone (BM, HAZ, and FZ) are showed in Table 3. Hardness was lower in the BM zone and higher in the FZ zone for both samples B and C. In sample A, the lowest hardness was obtained in the HAZ zone. In general, the highest hardness values were found in sample C and the lowest in sample A. The greatest difference in hardness between the samples was found in the HAZ zone.

**Figure 2:**
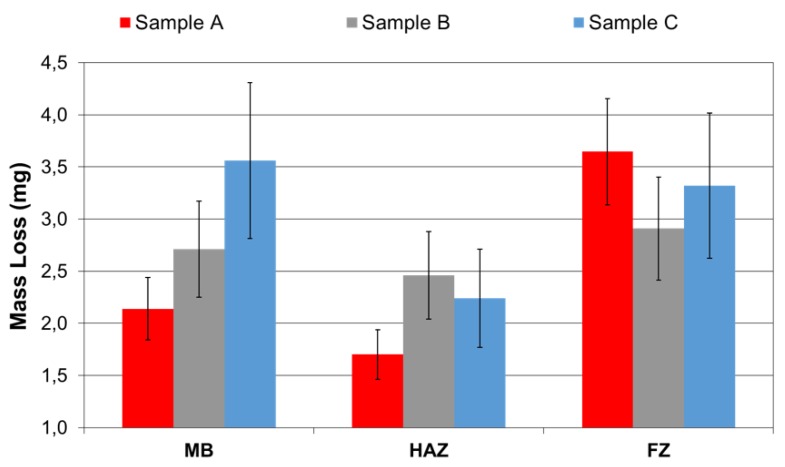
Mass loss in the sample zones.

The loss of mass in each zone after the pin on disk test is shown in Figure 2. This is lower in sample A, which indicates less wear, and higher in sample C. In these two samples, very different mass loss values were obtained, depending the zone analyzed. Sample C had higher hardness than sample A so that the loss of mass should have been lower. However, it was easier for high hardness particles to be released from sample C because their microstructure is more heterogeneous. These particles can act as abrasives during the rest of the test.

For sample A, the difference between the areas of major and minor mass loss was 110% and for sample C it was over 60%. These differences are not so high in the case of sample B, offering differences in mass loss of less than 20%. The consequence of a greater difference in the loss of mass is a less uniform profile of the inner pipe. Therefore, the flow of the steam circulating inside it becomes more turbulent, which causes greater corrosion [22,23,24,25]. Great turbulence favors the dissolution of the oxide layer, leaving the surface of the steel exposed to fluid. The underlying metal corrodes to form the oxide layer that is again removed by the liquid, and thus, the metal loss goes on [26,27,28,29,30]. The coefficient of friction measured for each sample in the different zones does not show significant variations, and reaches an average value around 0.65 in all cases. 

**Figure 3:**
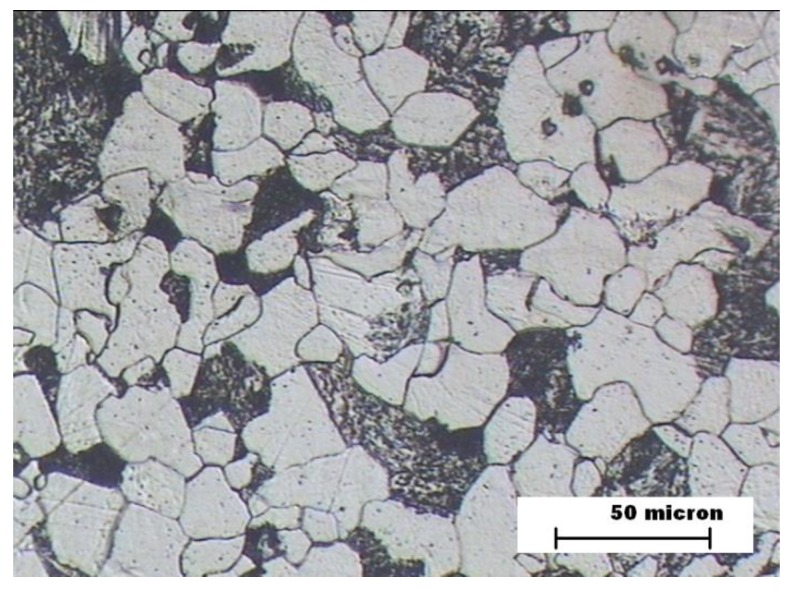
Samples A, B, and C. Microstructure of the steel (base metal—BM).

The proeutectoid ferrite formation was likely in the three welding zones as a result of the low carbon content of ASTM A335 hypoeutectoid steel [31,32]. The metallographic analysis of BM zone showed a microstructure formed by ferrite and perlite (Figure 3) in the three samples, since that zone is not thermally affected by a welding joint. An equivalent carbon of 0.5% was obtained, which makes the appearance of martensite very difficult due to the thermal effect of the welding process. 

**Figure 4:**
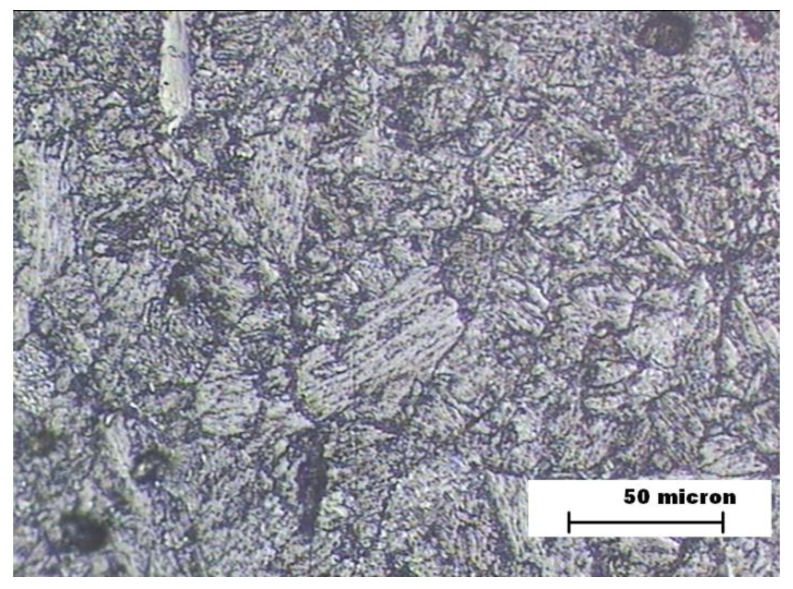
Sample A. Heat affected zone (HAZ) microstructure.

**Figure 5:**
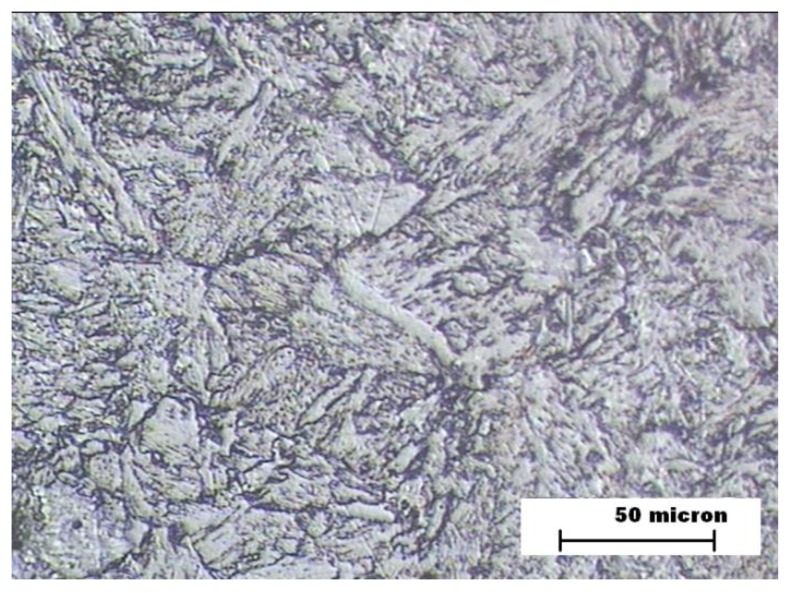
Sample A. Fusion zone (FZ) microstructure.

Figure 4 represents the microstructure of HAZ zone in the sample A. In this image, the presence of bainite is appreciated, which justifies the increase in wear resistance with respect to BM. Figure 5 shows the microstructure of FZ zone in the sample A. It consists of the columnar structure of the filler metal with the formation of narrow bands of proeutectoid ferrite on the grain boundary. This microstructure is the cause of the increase in hardness with respect to the other zones. The worse behavior against abrasive wear is explained by the presence of phases with different hardnesses [33,34].

**Figure 6:**
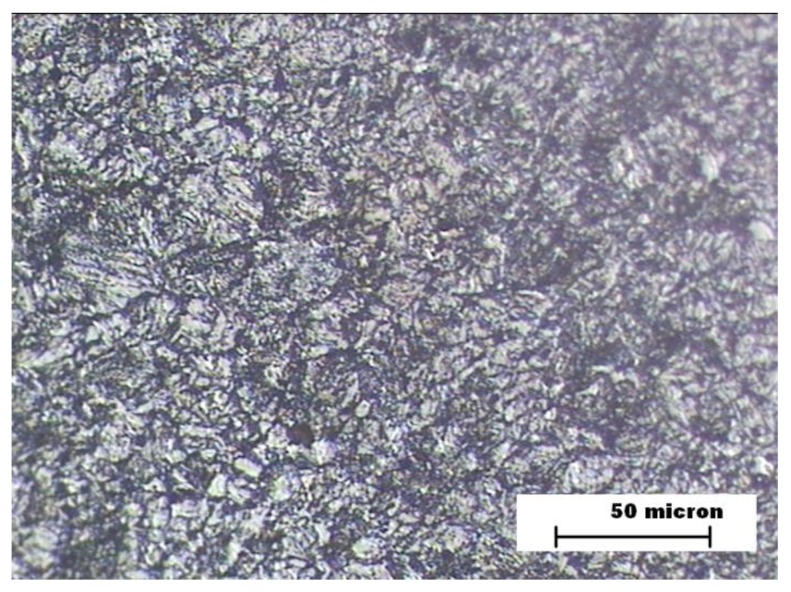
Sample B. HAZ microstructure.

**Figure 7:**
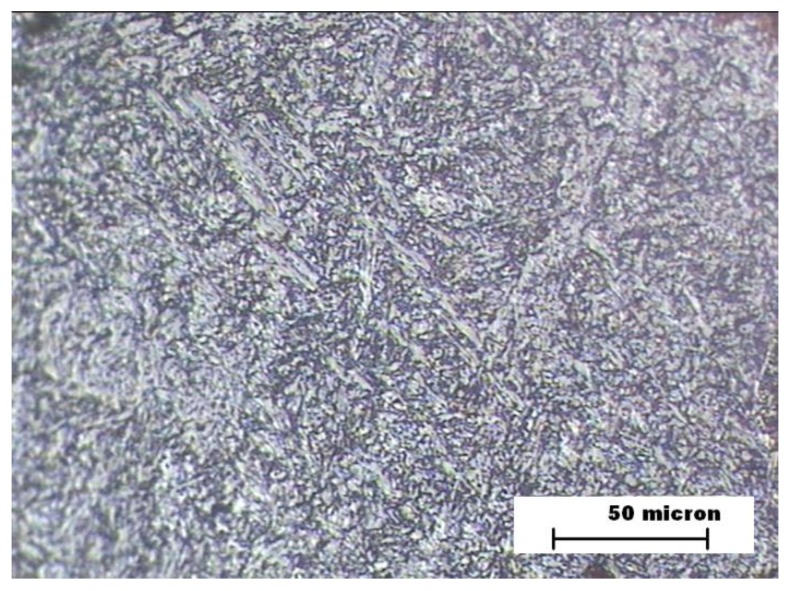
Sample B. FZ microstructure.

Figure 6 and Figure 7 show the images of the microstructures of the HAZ and FZ zones in the sample B. The sample HAZ microstructure includes a small proportion of ferrite and its grain size is smaller than the sample A HAZ. Hardness is higher than the sample B BM zone and much higher than sample A’s HAZ [35]. The wear resistance of B’s HAZ is worse than that observed for sample A’s HAZ due to the presence of ferrite. The microstructure of sample B’s FZ shows the presence of bainite in the form of needles accompanied by bands of filler metal and proeutectoid ferrite, which gives a similar hardness to the HAZ of the same sample.

**Figure 8:**
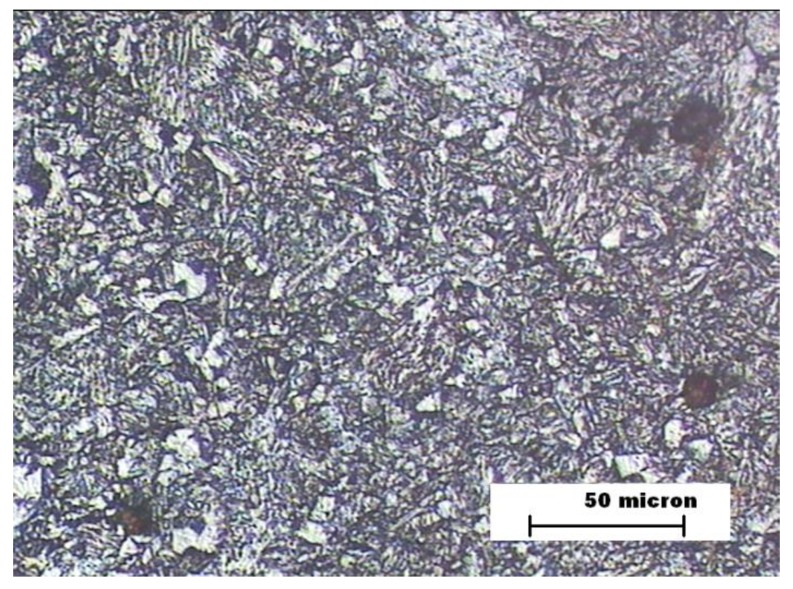
Sample C. HAZ microstructure.

**Figure 9:**
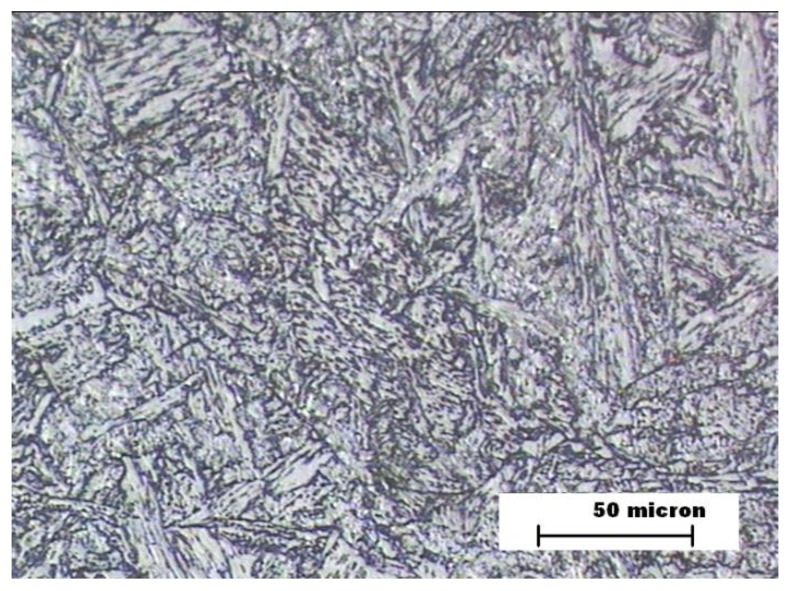
Sample C. FZ microstructure.

The microstructures for the HAZ and FZ zones of sample C are shown in Figure 8 and Figure 9 respectively. In the former’s case, the HAZ zone is characterized by the presence of bainite with dispersed ferrite, while the FZ zone consists of bands of the filler metal and proeutectoid ferrite. Those microstructures are responsible for the high hardness values of these zones that can be seen in Table 3. The wear behavior is similar in the HAZ and worse in the FZ with respect to sample B.

In summary, the hardness test shows that the highest values was found in the welded sample without the preheating process and post-welding heat treatment (sample C), and the lowest in the sample with both treatments (sample A). The metallographic analysis confirms this behavior.

The wear tests indicate that HAZ is the zone with the least weight loss, particularly in sample A, which in contrast, presents greater wear in the FZ zone. The wear of sample C was very high in the FZ and BM zones. On the other hand, the wear between zones was more uniform for sample B. The advantage presented by this uniformity is that it allows one to conclude that the geometry of the wear is favorable to the reduction of the turbulence, and therefore, to the erosion–corrosion phenomenon.

## 4. Conclusions

For this article, the abrasive wear resistance of three welded joints with different welding procedures was studied. For that purpose, pin on disk tests were carried out in three zones of each weld: BM, HAZ, and FZ. In addition, hardness was determined and a metallographic analysis of each zone was performed.

The sample that showed the lowest mass loss was A; however, the variation in wear that can be seen between the different zones (MB, BEAM, and FZ), generates an irregular profile on the surface that favors the action of the FAC. The same trend was found for sample C, but with high values of mass loss in the different zones. However, sample B showed a more homogeneous loss of mass in all the zones, such that it seems to be the most appropriate in terms of its behavior against the FAC.

In summary, it can be established that the elimination of the PWHT step in the standard welding procedure improves the response to the FAC phenomenon.

## Figures and Tables

**Table 1:** Chemical composition in weight percent of the steel evaluated.

C (%)	S (%)	Mn (%)	Si (%)	Cr (%)	Mo (%)
0.14	0.004	0.30	0.78	1.2	0.5

**Table 2:** Welding parameters.

Filler Material		TIG Rod Ø 2.4 mm	Shielded Metal Arc Welding					
			Electrode Ø 3.25 mm AWS A-5.5/Class E8018-B2L			Electrode Ø 2.5 mm AWS A-5.5/Class E8018-B2L		
Sample A	Temperature between Passes (°C)				50–55			
	Time (‘)	90	45	50	40	30	40	30
Sample B	Temperature between Passes (°C)	150	160	170	180	180	180	180
	Time (‘)	120	45	50	40	30	40	30
Sample C	Temperature between Passes (°C)				180–200			
	Time (‘)	90	45	45	45	30	35	30

**Table 3:** Hardness for the different regions of the joint.

Hardness (HV)			
Welding Process	BM	HAZ	FZ
Sample A	202	183	239
Sample B	212	233	256
Sample C	234	312	306
